# Capecitabine Versus Active Monitoring in Stable or Responding Metastatic Colorectal Cancer After 16 Weeks of First-Line Therapy: Results of the Randomized FOCUS4-N Trial

**DOI:** 10.1200/JCO.21.01436

**Published:** 2021-09-13

**Authors:** Richard A. Adams, David J. Fisher, Janet Graham, Jenny F. Seligmann, Matthew Seymour, Richard Kaplan, Emma Yates, Mahesh Parmar, Susan D. Richman, Philip Quirke, Rachel Butler, Ewan Brown, Fiona Collinson, Stephen Falk, Harpreet Wasan, Kai-Keen Shiu, Gary Middleton, Leslie Samuel, Richard H. Wilson, Louise C. Brown, Timothy S. Maughan

**Affiliations:** ^1^ Centre for Trials Research Cardiff University and Velindre NHS Trust, Cardiff, United Kingdom; ^2^ MRC Clinical Trials Unit at UCL, London, United Kingdom; ^3^ University of Glasgow and Beatson West of Scotland Cancer Centre, Glasgow, United Kingdom; ^4^ Leeds Institute of Medical Research, University of Leeds, Leeds, United Kingdom; ^5^ Bristol Genetics Laboratory, Bristol, United Kingdom; ^6^ Western General Hospital, Edinburgh, United Kingdom; ^7^ Bristol Cancer Institute, Bristol, United Kingdom; ^8^ Hammersmith Hospital London, London, United Kingdom; ^9^ University College Hospital London, London, United Kingdom; ^10^ University of Birmingham, Birmingham, United Kingdom; ^11^ Aberdeen Royal Infirmary, Aberdeen, United Kingdom; ^12^ MRC Oxford Institute for Radiation Oncology, Department of Oncology, University of Oxford, Oxford, United Kingdom

## Abstract

Despite extensive randomized evidence supporting the use of treatment breaks in metastatic colorectal cancer (mCRC), they are not universally offered to patients despite improvements in quality of life without detriment to overall survival (OS). FOCUS4-N was set up to explore the impact of oral maintenance therapy in patients who are responding to first-line therapy.

**METHODS:**

FOCUS4 was a molecularly stratified trial program that registered patients with newly diagnosed mCRC. The FOCUS4-N trial was offered to patients in whom a targeted subtrial was unavailable or biomarker tests failed. Patients were randomly assigned using a 1:1 ratio between maintenance capecitabine and active monitoring (AM). The primary outcome was progression-free survival (PFS) with secondary outcomes including OS toxicity and tolerability.

**RESULTS:**

Between March 2014 and March 2020, 254 patients were randomly assigned (127 to capecitabine and 127 to AM) across 88 UK sites. Baseline characteristics were balanced. There was strong evidence of efficacy for PFS (hazard ratio = 0.40; 95% CI, 0.21 to 0.75; *P* < .0001), but no significant improvement in OS (hazard ratio, 0.93; 95% CI, 0.69 to 1.27; *P* = .66) was observed. Compliance with treatment was good, and toxicity from capecitabine versus AM was as expected with grade ≥ 2 fatigue (25% *v* 12%), diarrhea (23% *v* 13%), and hand-foot syndrome (26% *v* 3%). Quality of life showed little difference between the groups.

**CONCLUSION:**

Despite strong evidence of disease control with maintenance therapy, OS remains unaffected and FOCUS4-N provides additional evidence to support the use of treatment breaks as safe management alternatives for patients who are stable or responding to first-line treatment for mCRC. Capecitabine without bevacizumab may be used to extend PFS in the interval after 16 weeks of first-line therapy.

## INTRODUCTION

Treatment breaks in patients receiving palliative chemotherapy for metastatic colorectal cancer (mCRC) reduce toxicity burden and improve quality of life (QoL).^1^ However, current standards either mandate or recommend a strategy of continuing therapy, until progression or excess toxicity. Standard maintenance strategies in high-income countries favor combined oral capecitabine with intravenous bevacizumab once every 3 weeks,^2,3^ on the basis of the phase III CAIRO3^4^ and AIO-0207^5^ studies. Health economic evaluation of this approach has previously indicated a lack of cost-effectiveness driven by nonsignificant improvement in overall survival (OS) and high costs of intravenous bevacizumab (drug plus administration).^6^ Previous studies have evaluated a range of strategies to either completely stop therapy as a treatment holiday, reducing toxicities and hospital visits, or attenuate therapy, removing certain drugs as a maintenance therapy in comparison with historic standard-of-care continuation of maximum tolerated dose of treatment. Meta-analysis of these approaches overall shows no difference in OS.^7^ Notably, maintenance strategies, almost uniformly, demonstrate an improvement in progression-free survival (PFS), but at the expense of ongoing (though attenuated) toxicity and unending multiple hospital visits for intravenous therapy. In the FOCUS4-N trial (embedded within the FOCUS4 trial program, see the Data Supplement [online only]), we have explored the oral strategy of capecitabine only versus active monitoring (AM). This will allow us to study the potential impact on PFS, toxicity, and QoL, which will enable patients and clinicians to choose an optimum approach tailored to the individual.

**CONTEXT**

**Key Objective**
In patients with metastatic colorectal cancer, first-line systemic anticancer therapy (SACT) with palliative intent aims to extend overall survival (OS) while maintaining quality of life. Current guidelines recommend a maintenance strategy of oral capecitabine and bevacizumab in patients with disease control after 4-6 months of induction SACT. This is based upon improved progression-free survival (without evidence of OS benefit) compared with a complete treatment break with active monitoring (AM). FOCUS4-N aims to establish the impact of maintenance capecitabine monotherapy versus AM.
**Knowledge Generated**
These results demonstrate that capecitabine can double the time until return to induction SACT. However, patients may adopt an AM approach without detriment in OS and with less toxicity.
**Relevance**
FOCUS4-N provides information for patients and clinicians, which will assist decision making at the end of induction SACT. Capecitabine without intravenous bevacizumab is likely more cost-effective than the current recommended approach of capecitabine and bevacizumab.

The FOCUS4 trial program is a molecularly stratified umbrella platform trial (Data Supplement) that evaluated safety and efficacy of novel treatments in targeted biomarker subgroups within a phase II or III trial setting. The trial used adaptive statistical methodology that allowed the addition of new therapies and the dropping of ineffective ones and including a nonstratified comparison (FOCUS4-N) for patients in whom a molecularly stratified comparison was unavailable or the biomarker tests failed for their tumor tissue. In the Data Supplement, we describe the design and methods for patient registration and biomarker testing. In this article, we report the findings of FOCUS4-N, which tested the efficacy of capecitabine as a maintenance therapy versus AM in patients with mCRC.

## METHODS

### Trial Approvals, Patient Eligibility, and Recruitment

The trial and subsequent amendments were approved by the UK National Ethics Committee Oxford (reference 13/SC/0111) and by the relevant regulatory body MHRA (CTA# 20363/0400/001 and EudraCT# 2012-005111-12).

Patients age at least 18 years with newly diagnosed locally advanced or mCRC were eligible for registration in the FOCUS4 trial program (see the Data Supplement for details of FOCUS4 design and registration methods). Patients whose tumors had remained stable or responded to treatment according to their 16-week computed tomography (CT) were assessed for eligibility for the FOCUS4-N comparison. In addition to the registration eligibility criteria, patients were required to have a baseline randomly assigned CT scan performed within 4 weeks prerandomization; a minimum 3-week washout period between the last chemotherapy or biologic therapy dose and the first capecitabine dose; adequate renal (creatinine clearance > 50 mL/min) and liver function; and a WHO performance status of 0-2. Patients who were eligible for either FOCUS4-N or a molecularly stratified trial were offered entry into either and given the option of which study to participate in, followed by appropriate consent.

In the first phase of FOCUS4 between January 2014 and June 2017, patients with raised baseline platelet count (thrombocytosis) were considered ineligible on the basis of previous data from the COIN trial (which indicated a significant survival detriment in this patient group receiving an intermittent strategy).^1^ A subsequent individual patient data meta-analysis of phase II or III intermittent strategy trials did not confirm the observation from COIN.^8^ Thus, between June 2017 and March 2020, eligibility criteria were adapted, allowing inclusion of this patient group with thrombocytosis.

### Trial Procedures

Patients randomly assigned to capecitabine were asked to continue taking the drug until disease progression, death, or intolerable toxicity. Capecitabine was dosed according to standard guidelines, orally twice daily for 14 days followed by a 7-day rest period without capecitabine tablets.

Patient tumor status was assessed every 8 weeks by CT scan reviewed at the treating hospital site according to RECIST version 1.1.^9^ Toxicities and symptoms were assessed locally every 4 weeks from random assignment or start of treatment using NCI CTCAE (version 3.0). Patients were followed until progressive disease, at which point the patient was recommended to restart first-line systemic therapy.

Treatment was stopped for grade ≥ 3 toxic effects or persistent toxicities judged medically important or not tolerated by the patient, until the toxicity resolved to grade 1 or better. After stopping treatment, capecitabine could be reinitiated at a reduced dose. Any stoppage for ≥ 28 days was not permitted, with the patient discontinued from trial therapy but remaining under follow-up.

QoL data using EQ-5D were collected at random assignment, every 8 (7-9) weeks until progression, 4 weeks after end of trial treatment, 3 months after progression, and then every 6 months.

### Statistical Methods

#### Treatment allocation.

Patients were allocated to capecitabine or AM by a centrally managed telephone service at the MRC Clinical Trials Unit at University College London, using a 1:1 allocation ratio by minimization with a random element of 20%. Minimization factors were treating hospital site, primary tumor site (right colon, left colon, or rectum), WHO performance status (0, 1, or 2), 16-week CT scan result (stable disease and partial or complete response), number of metastatic sites (none, one, or two or more), and first-line therapy regimen (fluorouracil, capecitabine, or neither; both oxaliplatin and irinotecan, irinotecan only, or neither; and cetuximab or panitumumab, bevacizumab, or no monoclonal antibody).

#### Outcome measures.

The primary FOCUS4-N outcome was PFS, defined as time from random assignment to either disease progression (according to RECIST criteria) or death from any cause. Patients without a PFS event were censored at the time of their last recorded CT scan. OS was a secondary outcome, defined as time from random assignment to death from any cause with patients censored at last recorded disease assessment, blood measurement, or anticancer treatment. Other secondary outcomes included safety, toxicity, QoL, and tumor response. QoL was analyzed using mixed-effects linear modeling with patient-level random intercepts and time slopes, with differences by the treatment arm tested by evaluating the area under the curve from the model.

#### Sample size calculation.

The FOCUS4-N target sample size was calculated using the Analysis of Resources for Trials program implemented in Stata software. Given that the recruitment rate into FOCUS4-N was dependent on the availability of other molecular comparisons, failure of biomarker testing, or patient choice, exact recruitment figures were unknown at the trial commencement. Various scenarios were used to estimate the recruitment rate over 5 years, and we assumed a 4-month median PFS in the AM arm (on the basis of COIN trial data). A total of 644 patients (635 events) would provide 80% power of detecting a hazard ratio (HR) of 0.8 at the two-sided 5% significance level.

In March 2020, the COVID-19 pandemic resulted in temporary closure of FOCUS4 to new recruitment. Following Independent Data Monitoring Committee review and recommendation, a decision was taken to close recruitment permanently in April 2020 as trial funding was nearing its end. A previous review of the implications of reduced recruitment on the statistical power of FOCUS4-N had been considered by our funders who recommended that, despite reduced power, the trial should close in 2020 and report the data accrued up to that point. Furthermore, at analysis, it became clear that the observed HR was substantially more extreme than the target HR on which we based our original sample size.

#### Statistical analysis.

All analyses were performed according to a predefined statistical analysis plan agreed before database lock. We analyzed using Stata statistical software, version 16.1 (Stata Corporation, TX). The primary analysis was performed according to intention-to-treat with a secondary per-protocol analysis defined by patients who completed at least one cycle of trial treatment (≥ 28 days). Patients were censored according to the following criteria. For survival status, we censored patients on the date that they were last known to be alive, either via collection of prescription from their hospital pharmacy or attendance at a follow-up visit or CT scan. For PFS, we censored patients without progression on the date of the last CT scan showing no progression. For patients who died before any follow-up visit or CT scan, we used the date of death as the date of the event and assumed death without progression, provided that the death occurred within 3 months of random assignment or any previous scan confirming no progression.

Kaplan-Meier curves were used to present survival data and Cox regression modeling to estimate HRs between randomized groups. Unadjusted HRs and the ones adjusted for the stratification factors used to minimize patients into allocated groups (primary analysis) were estimated. A further analysis also adjusted for resection status, timing of metastatic disease, alkaline phosphatase, white blood cell count, age of tumor sample, and use of aspirin at baseline. Deviation from nonproportional hazards was assessed using regression of scaled Schoenfeld residuals against the log of time.

## RESULTS

### Recruitment and Compliance

**FIG 1.**
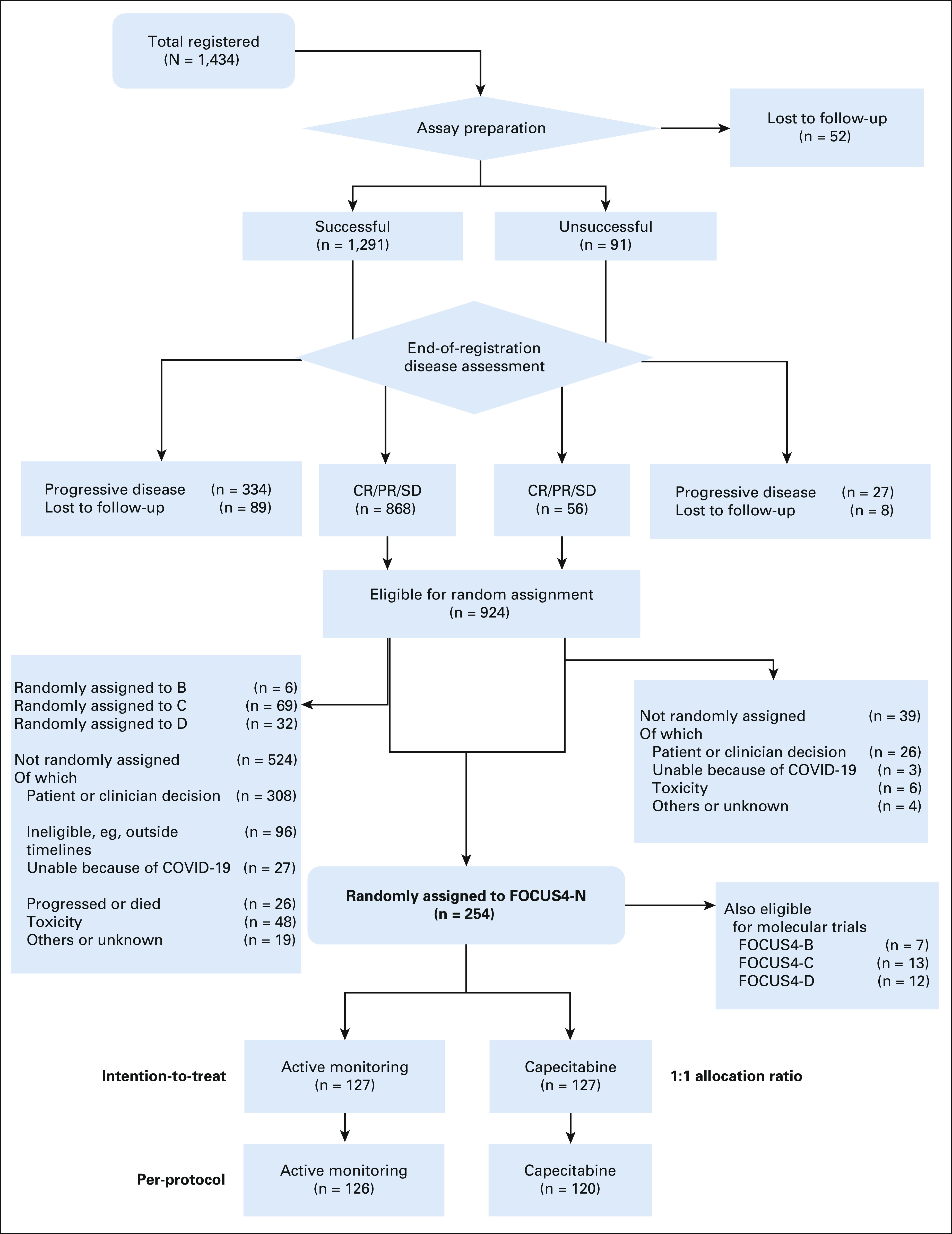
Flow diagram showing patient flow through the FOCUS4-N trial. CR, complete response; PR, partial response; SD, stable disease.

Across 88 UK hospitals, between January 2014 and March 2020, 1,434 patients were registered into FOCUS4, of whom 924 underwent successful biomarker assessment and completed 16 weeks of first-line therapy with either stable or responding disease (Data Supplement). Of these patients, 254 were randomly assigned to FOCUS4-N (Fig 1), 127 to AM and 127 to maintenance capecitabine. Baseline demographic and clinical characteristics were well-balanced between the study arms (Table 1 and Appendix Table A1, online only). Most patients had widespread synchronous metastatic disease with about half having an unresected primary tumor. A right-sided primary tumor location was present in about one third. The majority were treated with doublet chemotherapy (irinotecan-based 57%) without a monoclonal antibody (as bevacizumab is not reimbursed in the United Kingdom). The Data Supplement shows induction chemotherapy for all patients in FOCUS4, and the Data Supplement shows disease response to induction chemotherapy on the basis of biomarker subgroup. The molecular characteristics are shown in Table 1 (and the Data Supplement for all FOCUS4 participants), showing that only 37% had an *RAS* wild-type tumor reflecting NHS England policy of not allowing treatment breaks for patients on epidermal growth factor receptor monoclonal antibodies.

**TABLE 1.**
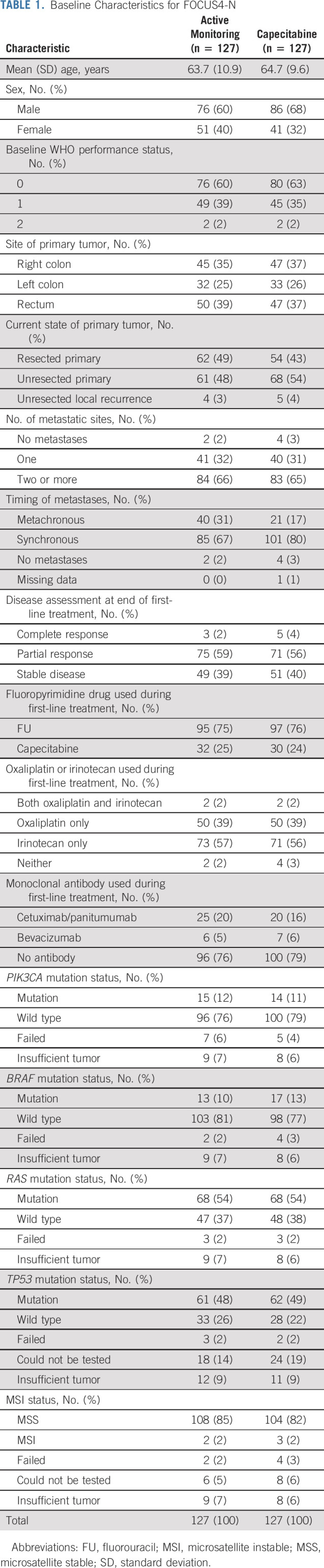
Baseline Characteristics for FOCUS4-N

Compliance with randomized allocation was good with only one patient in the AM arm receiving capecitabine approximately 6 months before progression. Patients in the capecitabine arm received a median of four cycles (interquartile range, 2-8).

### Primary Outcome: PFS

**FIG 2.**
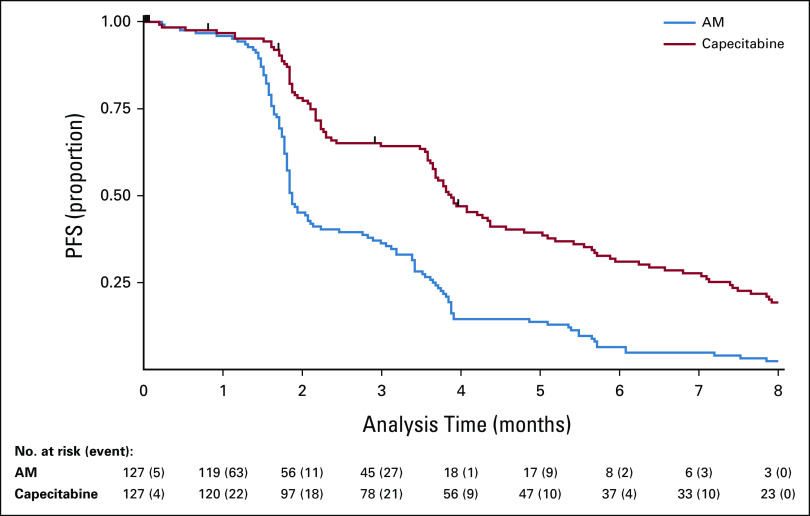
Kaplan-Meier curve for PFS in FOCUS4-N. Cox regression HR, adjusted for minimization factors = 0.40 (95% CI, 0.21 to 0.75), *P* < .0001. Minimization factors: location of primary tumor (left, right, and rectum), baseline WHO performance status, baseline disease assessment, No. of metastases, first-line therapy (fluoropyrimidine, oxaliplatin or irinotecan, and monoclonal antibody), and biomarker cohort, stratified for FOCUS4 trial timepoints. AM, active monitoring; HR, hazard ratio; PFS, progression-free survival.

There were 122 of 127 PFS events in the AM arm and 117 of 127 in the capecitabine arm. The median PFS in the capecitabine arm was 3.88 months (95% CI, 3.65 to 4.37) and 1.87 months (95% CI, 1.81 to 2.14) in the AM arm. Unadjusted and adjusted HRs were 0.44 (95% CI, 0.33 to 0.57), *P* < .0001 and 0.40 (95% CI, 0.21 to 0.75), *P* < .0001, respectively. Figure 2 shows Kaplan-Meier curves. Per-protocol analyses demonstrated very similar findings; unadjusted and adjusted HRs were 0.42 (95% CI, 0.32 to 0.55), *P* < .0001 and 0.38 (95% CI, 0.28 to 0.51), *P* < .0001, respectively. There was no evidence to suggest deviation from the proportional hazards assumption (*P* = .084).

### OS

**FIG 3.**
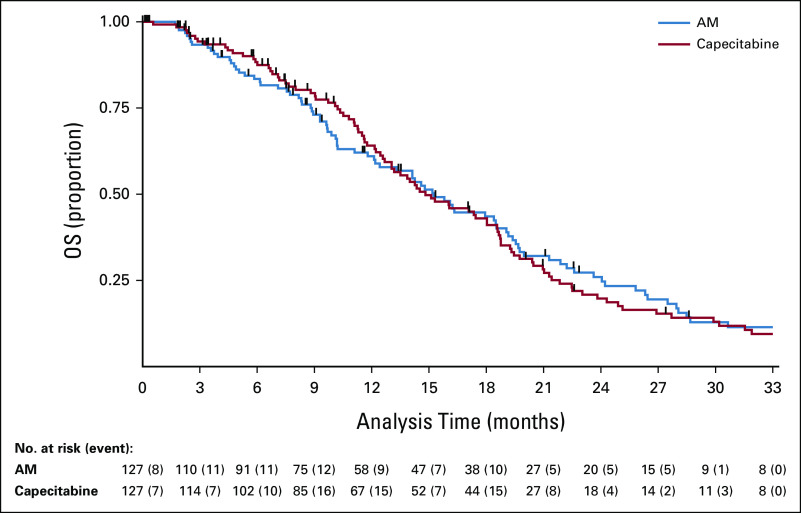
Kaplan-Meier curve for OS in FOCUS4-N. Cox regression HR, adjusted for minimization factors = 0.93 (95% CI, 0.69 to 1.27), *P* = .66. Minimization factors: location of primary tumor (left, right, and rectum); baseline WHO performance status; baseline disease assessment; No. of metastases; first-line therapy (fluoropyrimidine, oxaliplatin or irinotecan, and monoclonal antibody); and biomarker cohort, stratified for FOCUS4 trial timepoints. AM, active monitoring; HR, hazard ratio; OS, overall survival.

There were 90 of 127 deaths in the AM arm and 99 of 127 deaths in the capecitabine arm. The median time to death was 15.2 months (95% CI, 12.1 to 18.5) in the AM arm versus 14.8 months (95% CI, 23.7 to 18.6) in the capecitabine arm, with no survival difference between the arms; unadjusted and adjusted HRs were 1.00 (95% CI, 0.75 to 1.33), *P* = .98 and 0.93 (95% CI, 0.69 to 1.27), *P* = .66, respectively. Kaplan-Meier curves are presented in Figure 3. There was no evidence to suggest deviation from the proportional hazards assumption (*P* = .58).

### Subgroup Analyses

**FIG 4.**
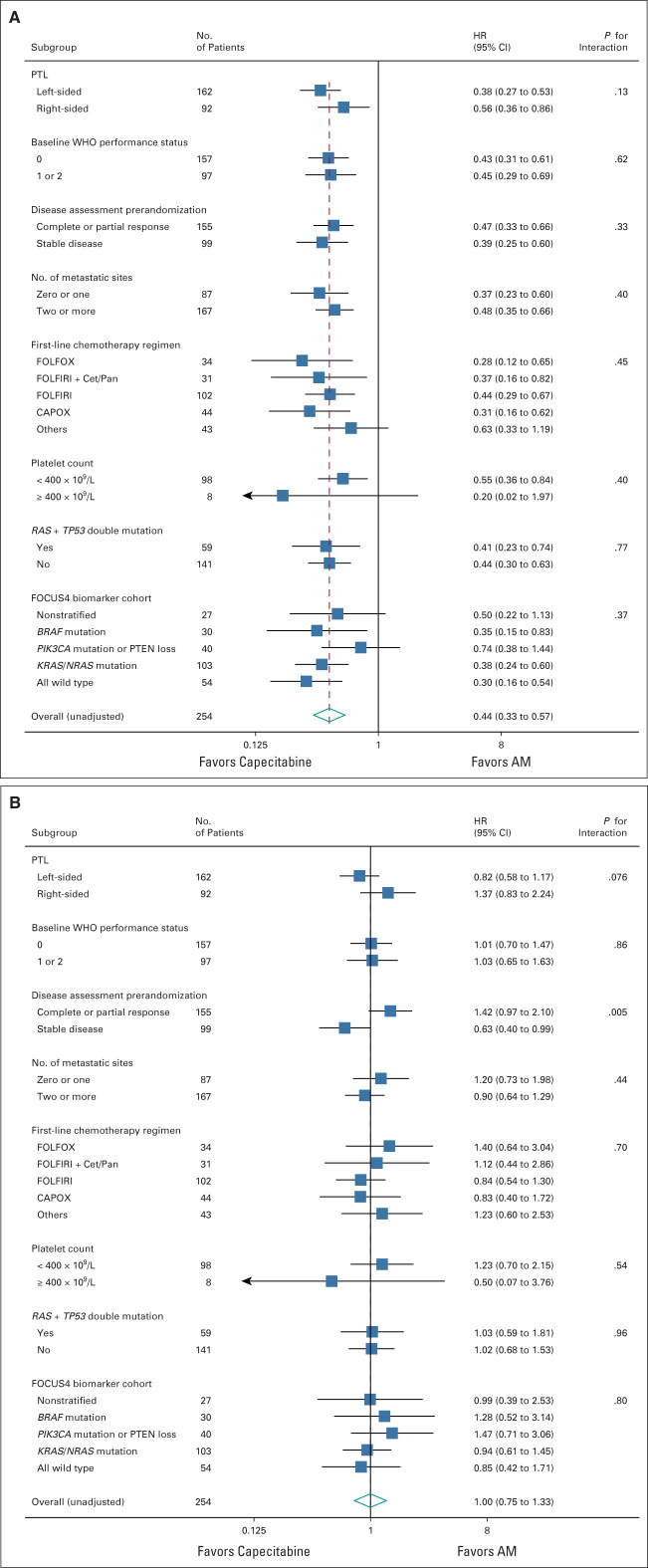
(A) Forest plot of subgroup analyses for PFS (unadjusted HRs). (B) Forest plot of subgroup analyses for OS (unadjusted HRs). AM, active monitoring; CAPOX, capeciteabine with oxaliplatin; Cet, cetuximab; FOLFIRI, fluorouracil, leucovorin, and irinotecan; FOLFOX, infusional fluorouracil, leucovorin, and oxaliplatin; HR, hazard ratio; OS, overall survival; Pan, panitumumab; PFS, progression-free survival; PTEN, phosphatase and tensin homolog; PTL, primary tumor location.

**FIG A1.**
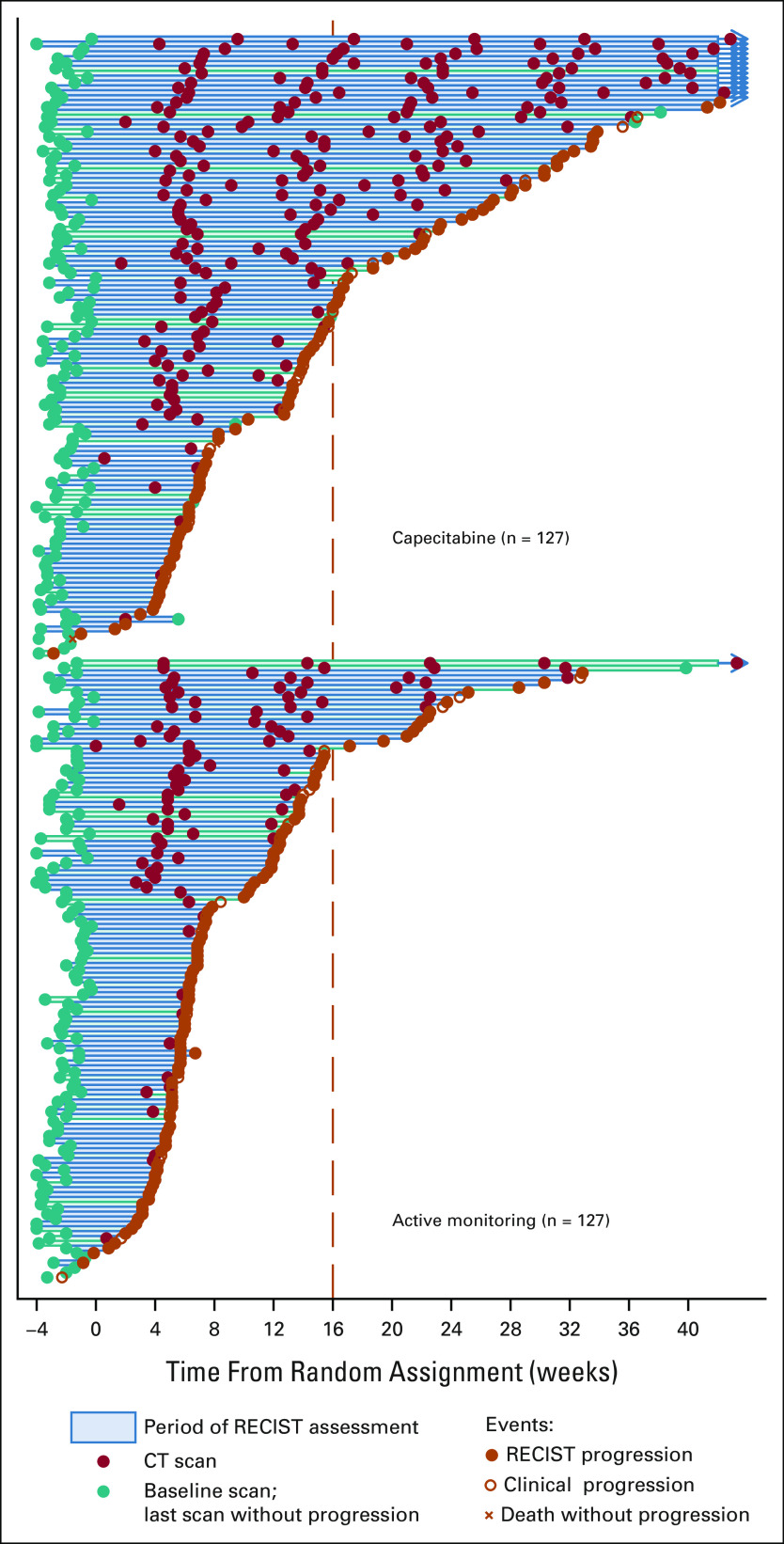
Swimmer plot for FOCUS4-N, by location of primary tumor. CT, computed tomography.

Preplanned subgroup analysis for PFS (Fig 4A) suggested better PFS with a maintenance strategy in left-sided tumors (HR 0.38 *v* 0.56 for right-sided, interaction *P* = .13), and a similar observation was seen with OS (HR 0.82 for left-sided *v* 1.37 for right-sided, interaction *P* = .076; Fig 4B). There was a suggestion that patients with tumoral loss of phosphatase and tensin homolog and *PIK3CA* mutations may show less benefit from maintenance capecitabine than other molecular subgroups (PFS HR 0.74, OS HR 1.47), although this was not statistically significant. For OS, the only other notable subgroup effect was that those with stable disease at random assignment appeared to benefit from maintenance capecitabine, whereas those with partial response did not (OS HR 0.63 and 1.42, respectively, interaction *P* = .005; Fig 4B). Swimmer plots show the distribution of individual patient PFS duration and timing of CT scans by left- versus right-sided disease (Appendix Fig A1, online only).

### Toxicity

**FIG 5.**
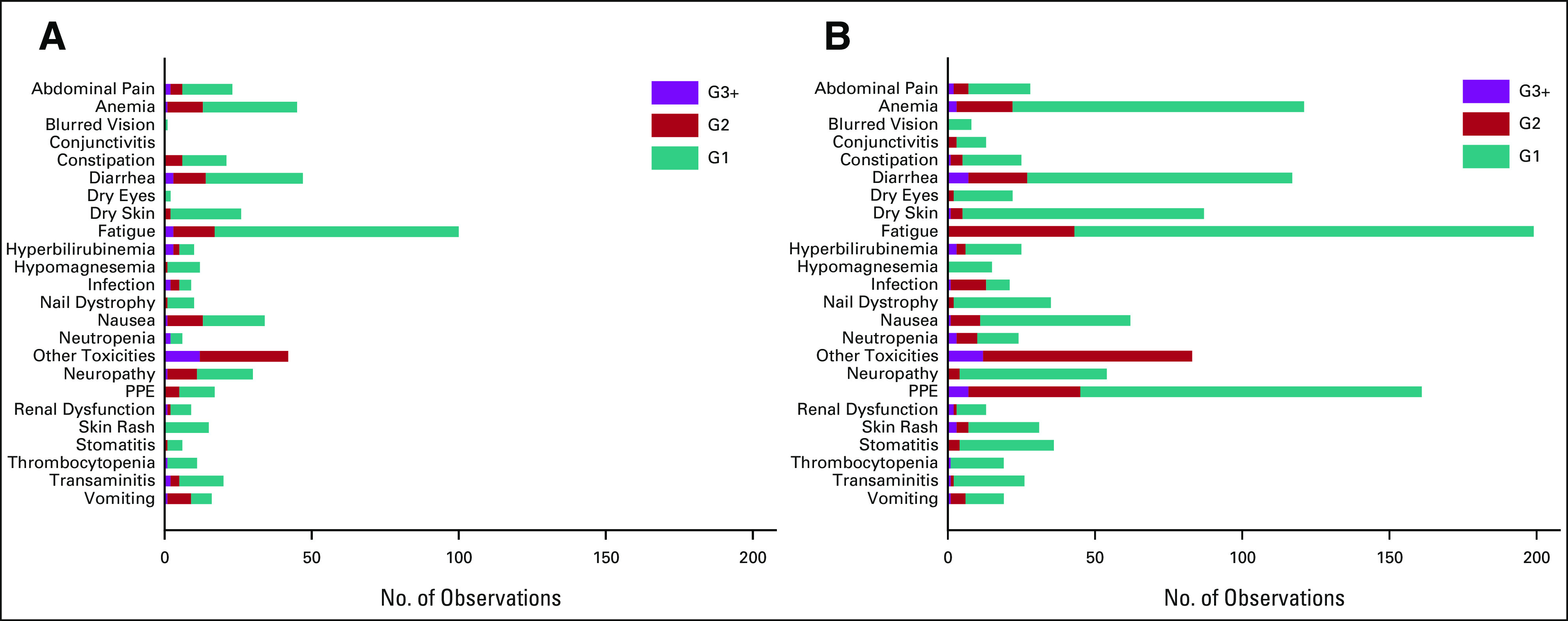
Cumulative reported toxicity by randomized group: (A) active monitoring (n = 127) and (B) capecitabine (n = 127). G, grade; PPE, palmar-plantar erythema.

Cumulative toxicities were substantially less in the AM arm, with increased toxicities associated with capecitabine maintenance including diarrhea, dry skin, fatigue, nausea, and palmar-plantar erythema (PPE; Fig 5). Ideally, a maintenance therapy should result in no toxicity. Incidence of grade zero as the worst toxicity reported per patient is therefore instructive and is as follows for AM and capecitabine maintenance, respectively: nausea 74% versus 67%, diarrhea 72% versus 46%, stomatitis 90% versus 77%, dry skin 83% versus 77%, PPE 87% versus 44%, and anemia 69% versus 54% (Appendix Table A2, online only).

During the trial, 51% of patients who received capecitabine had at least one cycle delayed, 37% had a dose reduction, and 34% missed at least one dose (within a cycle). Fifty percent of capecitabine patients commenced at least four cycles, and 25% commenced at least eight cycles.

### QoL

**FIG 6.**
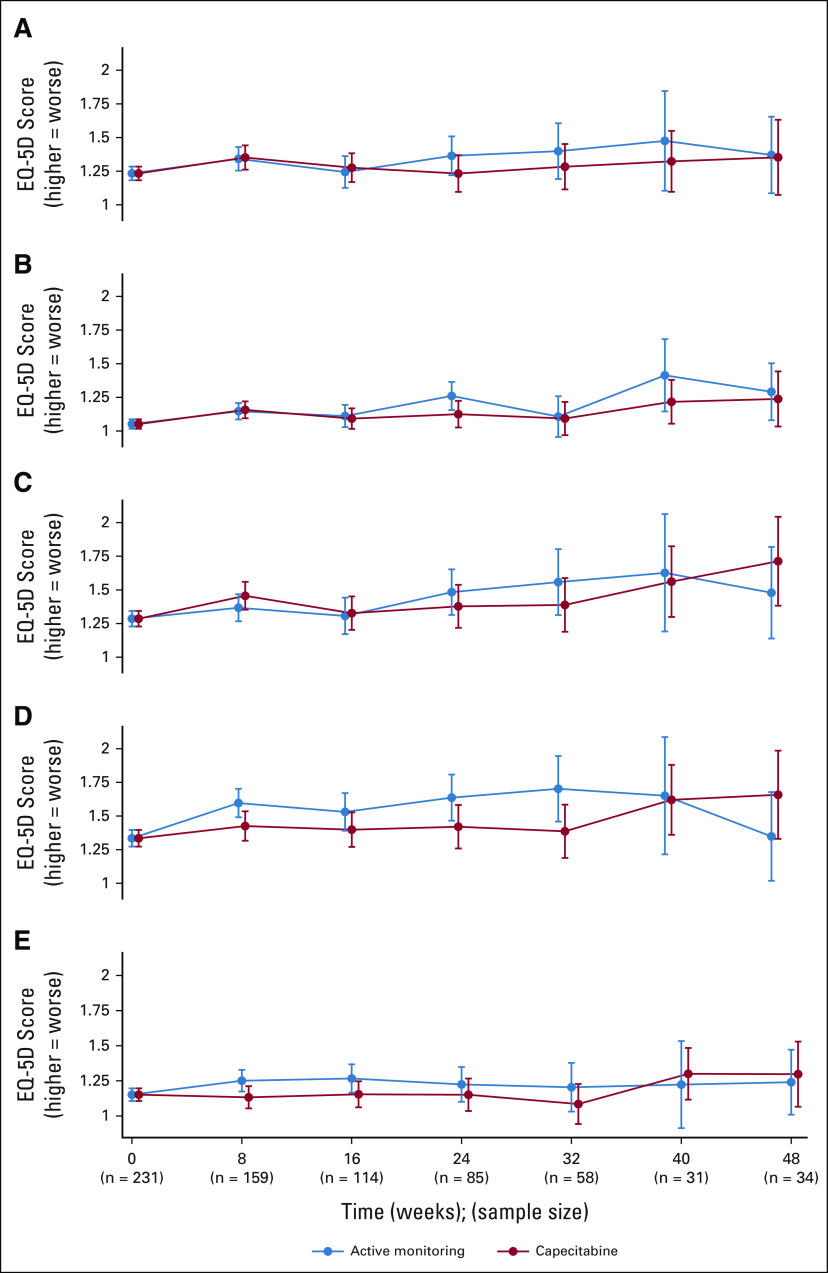
Quality of life measured by EQ-5D by randomized group: (A) mobility: X^2^ for AUC difference = 0.86(1), *P* = .35; (B) self-care: X^2^ for AUC difference = 1.64(1), *P* = .20; (C) usual activities: X^2^ for AUC difference = 0.06(1), *P* = .81; (D) pain and discomfort: X^2^ for AUC difference = 2.49(1), *P* = .11; and (E) anxiety and depression: X^2^ for AUC difference = 1.03(1), *P* = .31; AUC, area under the curve.

EQ-5D forms were completed in 93% (AM) and 90% (capecitabine) at baseline (prerandomization but postinduction chemotherapy). The Protocol (online only) mandated completion every 8 weeks until progression and 6-monthly thereafter; for analysis purposes, all available forms were forced into an 8-week schedule. On this basis, 63%, 45%, and 33% of randomly assigned patients had data available at 8, 16, and 24 weeks, respectively, with continuous decline thereafter. Modeling was applied to data up to 48 weeks, since data became too sparse beyond this. No notable differences were seen in mobility, self-care, usual activities, anxiety, and depression. There was a suggestion that pain and discomfort might have been experienced less within the capecitabine maintenance arm (*P* = .11, Fig 6). This may be due to symptoms associated with increased rates of progression in the AM arm.

## DISCUSSION

Choices on how to proceed with palliative treatment, in the large majority of patients with incurable mCRC, with stable or responding disease after 16 weeks of first-line therapy need careful consideration with the patient at the core. Discussions must be informed by the impact of receiving systemic anticancer therapy over the preceding period. This should include evaluation of the burden of toxicity and QoL, as well as the response to treatment. Pooled data from key phase II and III trials suggest minimal impact on OS from a maintenance or continuation strategy but do show the ability to delay a return to full combination therapy by implementation of a maintenance therapy. Notably, the FOCUS4-N data support the use of an oral only therapy (capecitabine) to extend PFS and delay a return to combination therapy by an average of two months. There is a clear cost to the patient for this improved PFS seen with maintenance capecitabine including worse toxicity in terms of diarrhea, fatigue, nausea, skin rash, and PPE albeit mostly at grade ≤ 2, and these factors should be used to further inform decision making. There was no difference in QoL scores between the two arms. It is notable that the swimmer plots (Appendix Fig A1) suggest that about a third of patients experience an extended PFS beyond 16 weeks with maintenance capecitabine, suggesting significant fluoropyrimidine sensitivity, while a third of patients demonstrate relative insensitivity to fluoropyrimidine monotherapy and may indicate a further need to explore predictive biomarkers of efficacy for this strategy. Preplanned subgroup analysis suggests that patients with stable disease at the end of 16-week induction period may gain a significant survival benefit from maintenance capecitabine, but this is not corroborated in other studies where the same phenomenon was assessed.^8^

Although this trial is underpowered to evaluate OS, it demonstrates very similar median values of 14.8 versus 15.2 months between the two arms with an HR of 0.93 (*P* = .66) when adjusted for minimization factors. It is informative to compare these data with those of CAIRO3, which compared an AM strategy with capecitabine plus bevacizumab maintenance with comparable effects on PFS (HR = 0.40, *P* < .0001; cfFOCUS4-N adjusted HR = 0.40, *P* < .0001) and nonsignificant OS effect (HR = 0.83, *P* = .06).^4^ Cross-trial comparisons carry notable caveats and must be undertaken with caution as CAIRO3 included patients with better prognosis than FOCUS4-N and both their median PFS and cycle number on maintenance therapy were approximately double those of ours. However, it does suggest that the main driver of PFS improvement when using capecitabine plus bevacizumab is the capecitabine. Individual patient data meta-analysis has also shown no OS benefit from current maintenance therapy strategies.^8^

On the basis of a subgroup analysis from the much larger phase III COIN study,^1^ which demonstrated a survival detriment in patients with a baseline thrombocytosis receiving a complete treatment break (HR = 1.55; *P* = .0018), we elected not to recruit patients with baseline thrombocytosis to the FOCUS4 trial program from January 2014 to June 2017. Wishing to validate or refute this finding, we undertook an individual patient data meta-analysis to assess thrombocytosis as a predictive marker of the benefits or otherwise of an intermittent or continuous therapy strategy.^8^ This evaluation did not validate our COIN finding on thrombocytosis, and thus, trial eligibility was adapted to allow these patients to enroll. Within FOCUS4-N overall, 3% (n = 8) of patients had baseline thrombocytosis, and thus, our study is underpowered to explore this predictive phenomenon further. Because of our conservative approach, FOCUS4-N under-represents approximately 25% of patients with mCRC who typically have thrombocytosis at baseline, a known worse prognosis group. However, given our findings in the individual patient data meta-analysis, we do not feel that this undermines our more general conclusions, which are independent of baseline platelet count.

Owing to funding restrictions in the UK National Health Service, bevacizumab is not routinely available for patients with mCRC, and in patients with *RA*S wild-type tumors, epidermal growth factor receptor monoclonal antibodies are only available in the first-line setting, with restrictions in England preventing treatment interruption of cetuximab/panitumumab for longer than 6 weeks. Additionally, during the FOCUS4-D trial recruitment period,^10^ patients with *RAS* wild-type and *BRAF* wild-type tumors were eligible for random assignment and were preferentially recruited into that trial. These factors make for a selective group of patients recruited to FOCUS4-N during that time. From a molecular perspective, 59% of patients randomly assigned in the FOCUS4-N trial had an *RAS* mutation and 15% a *BRAF* mutation. Reassuringly, the Forest plots (Figs 4A and 4B) do not show any significant differences in PFS or OS on the basis of these molecular criteria.

In conclusion, despite strong evidence of disease control with maintenance therapy, OS remains unaffected and FOCUS4-N provides additional evidence to support the use of treatment breaks as safe management alternatives for patients entering treatment de-escalation after 16 weeks of induction therapy for mCRC. If maintenance therapy is selected following consideration of the advantages and disadvantages in consultation with a particular patient, capecitabine without bevacizumab may be used to extend PFS, in the interval after doublet or triplet therapy, essentially doubling the period before recommencing full-dose induction therapy. Notably, these data also provide tools to best inform the dialogue between patients and clinicians on the pros and cons of the different approaches and their trade-offs.

## Figures and Tables

**Table:**
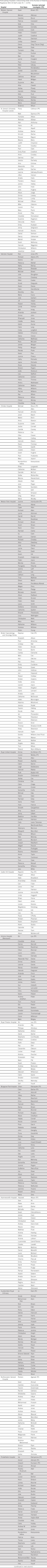
Participating Hospitals in Descending Order of the Number of Patients Registered With All Staff Listed (N = 2,076)

**TABLE A1.**
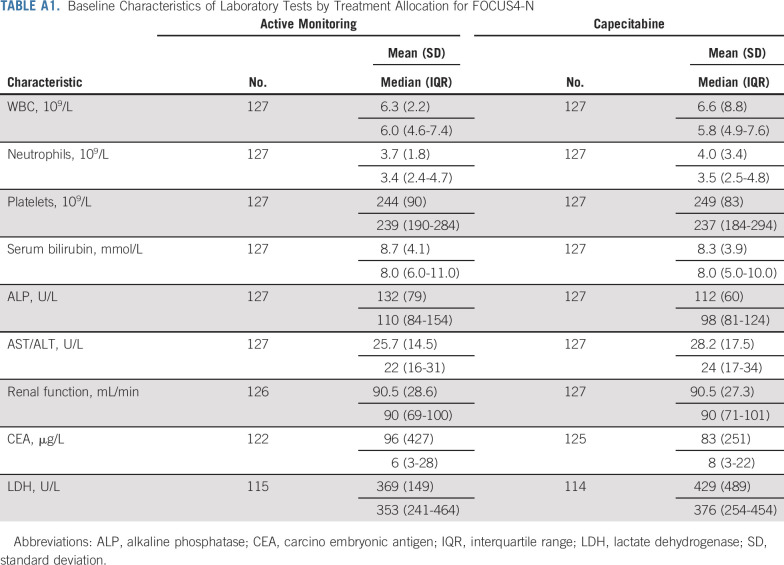
Baseline Characteristics of Laboratory Tests by Treatment Allocation for FOCUS4-N

**TABLE A2.**
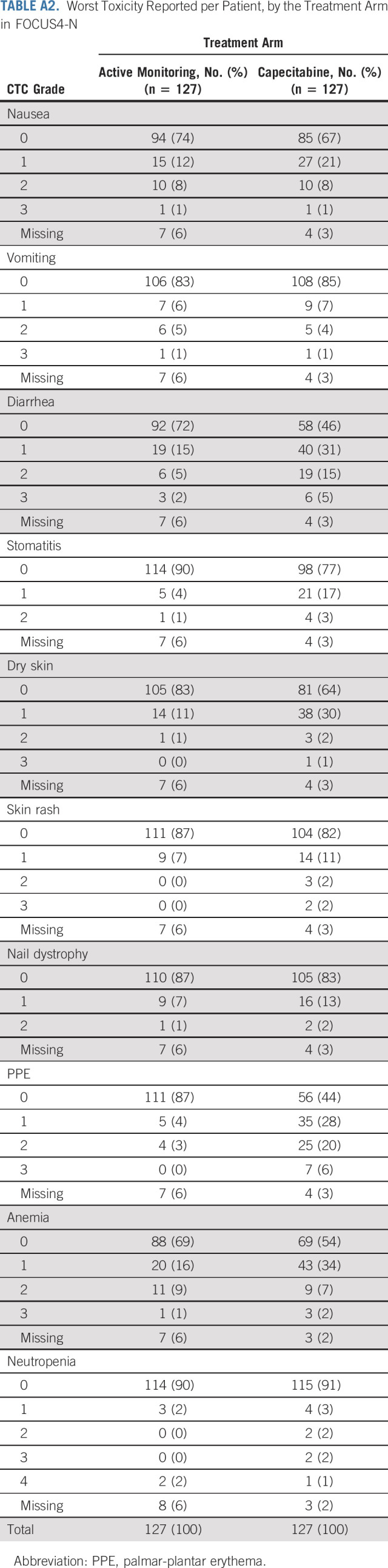
Worst Toxicity Reported per Patient, by the Treatment Arm in FOCUS4-N

## Data Availability

Individual deidentified participant data (including data dictionaries) can be shared upon appropriate application to the MRC CTU at any time from full publication. Study protocols and statistical analysis plan have been provided in the Data Supplement with this manuscript. Going forward, it is proposed that data will be shared with an appropriate international collaborative repository to enable future IPD meta-analysis.

## APPENDIX 1. Full Authorship List for the FOCUS4 Trial Investigators

### Writing Committee for FOCUS4-N Trial

Adams R., Fisher D.J., Graham J., Seligmann J.F., Seymour M., Kaplan R., Yates E., Parmar M., Richman S.D., Quirke P., Butler R., Brown E., Collinson F., Falk S., Wasan H., Shiu K., Middleton G., Samuel L., Wilson R.H., Brown L.C., Maughan T.S.

### Trial Management Group

Maughan T.S. (Chair), Wilson R., Adams R., Seymour M., Seligmann J.F., Graham J., Wasan H., Pope M., Pope J., Samuel L., Shiu K., Church D., Middleton G., Steward W., Twelves C., Wellman S., Hodgkinson E., Stoner N., Beety J., Duggleby K., Dutton G., MRC CTU team (below), laboratory staff (see below).

### MRC CTU trial coordinating centre

Brown L.C., Kaplan R., Parmar M., Fisher D.J., Campos M., Yates E., Santana S., Fiddament A., Harper L., Bara A., Pugh C., Bathia R., Letchemanan K., Przybył B., Bhogal S., Gopalakrishnan G., Purvis C., Diaz-Montana C., Mohamed F., Townsend S., Cragg W., Masters L., Van Looy N., Rauchenberger M.

### Laboratories

Quirke P., Richman S.D., Hemmings G., Davis J., Gallop N., Wilkinson L., Butler R., Roberts H., Jasani B., White R., Dodds R., James M., Morgan M.

### Independent Data Monitoring Committee

Cameron D. (Chair), Souhami R., Peeters M., Billingham C., Griffiths G., Brown J.

### Trial Steering Committee

Johnson P. (Chair), Rudd R., Whelan J., Russell A.
